# Mechanisms driving phenological and range change in migratory species

**DOI:** 10.1098/rstb.2018.0047

**Published:** 2019-07-29

**Authors:** Jennifer A. Gill, José A. Alves, Tómas G. Gunnarsson

**Affiliations:** 1 School of Biological Sciences, University of East Anglia, Norwich Research Park, Norwich NR4 7TJ, UK; 2 Department of Biology and CESAM—Centre for Environmental and Marine Studies, University of Aveiro, Campus de Santiago, 3810-193 Aveiro, Portugal; 3 South Iceland Research Centre, University of Iceland, Lindarbraut 4, 840 Laugarvatn, Iceland

**Keywords:** avian, climate change, migration, mismatch

## Abstract

Many migratory systems are changing rapidly in space and time, and these changes present challenges for conservation. Changes in local abundance and site occupancy across species' ranges have raised concerns over the efficacy of the existing protected area networks, while changes in phenology can potentially create mismatches in the timing of annual events with the availability of key resources. These changes could arise either through individuals shifting in space and time or through generational shifts in the frequency of individuals using different locations or on differing migratory schedules. Using a long-term study of a migratory shorebird in which individuals have been tracked through a period of range expansion and phenological change, we show that these changes occur through generational shifts in spatial and phenological distributions, and that individuals are highly consistent in space and time. Predictions of future rates of changes in range size and phenology, and their implications for species conservation, will require an understanding of the processes that can drive generational shifts. We therefore explore the developmental, demographic and environmental processes that could influence generational shifts in phenology and distribution, and the studies that will be needed to distinguish among these mechanisms of change.

This article is part of the theme issue ‘Linking behaviour to dynamics of populations and communities: application of novel approaches in behavioural ecology to conservation’.

## 1. Introduction

Migratory populations throughout the world are changing rapidly, with declines in abundance being reported on all major flyways [1–3] and driving international calls for action [4]. Identifying the causes of population changes in migratory species is inherently complex because of the range of sites and conditions experienced by individuals on their annual journeys. Changing conditions in any or all parts of migratory ranges could drive changes in abundance and distribution. For example, changes in local conditions could influence local demography and thus alter relative abundances and site occupancy anywhere within a migratory range. However, the effects of local changes in one part of a range can also cascade through to influence abundance and distribution across a range [5,6]. This potential for interactions between local environmental conditions, demography, individual development and range-wide distribution and phenology make migratory systems complex, and designing conservation actions to halt and reverse declines in migratory species is therefore a major challenge.

Recent changes in abundance of migratory species have been linked to their distribution and phenology. For example, phenological change is most commonly observed as shifts in the timing of migration to breeding grounds, and the magnitude of these shifts can vary greatly among species. Among European breeding birds, declines have been more frequent in species for which advances in spring arrival dates have not occurred [7], and species with non-overlapping breeding and wintering ranges are both more likely to be declining and less likely to have shown advances in spring arrival [2]. A lack of advance in spring arrival dates can potentially increase the impact of any trophic mismatch resulting from climate-driven changes in the timing of availability of key food resources for breeding [8,9]. Declining species with little or no phenological change are often assumed to be constrained from responding to changing climatic conditions in breeding areas (for example, because they migrate to more distant non-breeding locations), but the nature of any such constraint is unknown. Identifying the mechanisms through which phenological change occurs, and thus the factors constraining or facilitating these changes, may therefore be key to designing and targeting conservation actions to mitigate the effects of trophic mismatch.

Changes in the distribution of breeding and non-breeding ranges have also occurred in many migratory species, with polewards range shifts being particularly prevalent [10,11]. Concerns have consequently arisen over the efficacy of the existing protected area networks [5,12]. Range change is often interpreted as a response to changes in the suitability of environmental conditions (e.g. colonization of areas that were previously unsuitable and/or contraction from areas of declining suitability). However, the mechanisms driving changes in the distribution of individuals across a range are rarely known.

Both range change and phenological change could arise through individual plasticity in the use of space and time. For example, range change may occur through individual movement to locate suitable conditions, while changes in the timing of migration could arise through individual decisions on departure timings or time spent on migratory journeys varying between years. By contrast, these changes could also result from generational shifts in the frequency of individuals that use different locations or migrate at specific times. Generational shifts would not require individual plasticity but would require changes in the conditions determining the frequency of individuals within a population with different phenologies or probabilities of occupying different parts of a range, such that the spatial and temporal distribution of recruits to the population would differ from their predecessors. For example, changes in distribution could arise through shifts in the conditions influencing the probability of occupancy of different locations by new recruits, and/or by shifts in the survival rates achieved within different locations. Similarly, shifts in the timing of migration could occur through changes in the conditions influencing adoption of migration schedules by new recruits (or survival rates associated with different schedules) altering the frequency of individuals on different schedules within a population [13].

Individual plasticity in spatial distribution and migratory timings would facilitate relatively rapid responses to changing environmental conditions. However, generational shifts would likely result in slower responses to environmental changes, particularly in long-lived species, as the direction and magnitude of the changes would depend on the proportion of annual recruits within a population and the proportion of those recruits experiencing changing drivers of the use of space and time. If individual variation in migratory destination or timing has a genetic component, then generational shifts could drive microevolutionary change. However, genetic change is not an inevitable consequence of generational shifts, as individual destinations or timings could also be determined by environmental or social cues.

Changes in range size or migration phenology of populations as a result of individual plasticity in the use of space and time has not been demonstrated, and a growing number of individual tracking studies are reporting high levels of repeatability in individual migratory destinations and timings [13–20]. If generational shifts are the more likely driver of population changes in space and time, then our focus should be on understanding drivers of settlement and phenology of recruits, as well as their subsequent survival and recruitment.

Identifying the relative contributions of generational shifts and individual plasticity requires model systems in which individuals can be tracked across space and time, through periods of shifts in range and phenology. Such large-scale, long-term tracking data are rare but one system that provides all of these elements is the Icelandic black-tailed godwit, *Limosa limosa islandica*, which has been the subject of intensive individual and population studies since the mid-1990s [21–23]. In common with many migratory bird species at temperate and subarctic latitudes, advances in the phenology of spring migration have occurred in the Icelandic godwit population in recent decades [24]. Iceland supports very large breeding populations of several shorebird populations which migrate south to locations ranging from temperate Europe to sub-Saharan Africa [25]. The first recorded arrival dates of these species into south Iceland in spring have advanced over the last three decades, with godwits showing one of the most rapid advances (approx. two weeks earlier now than in the 1990s; [24]). In addition, this godwit population has shown sustained increases in number for over a century, likely as a consequence of warming conditions in Iceland facilitating earlier and more successful breeding and recruitment [6,26,27]. This population growth has been accompanied by range expansion in both the breeding and non-breeding ranges; in Iceland, godwits have expanded from a breeding range that was confined to the southwest corner of the country around 1900, to occupy progressively more northerly and easterly locations [27]. In the non-breeding range, which spans coastal areas of northwest Europe from Britain and Ireland to Iberia and northern Morocco, colonization and population increases have primarily occurred in the northern part of the range (east and northwest England, Scotland and east Ireland) since the 1970s, when surveys began [21,28].

In the mid-1990s, a programme of population-wide and lifelong tracking of individual godwits was initiated [21,29]. Across the breeding and non-breeding ranges, godwits have been caught and marked with unique combinations of coloured leg-rings, and approximately 1–2% of the population (which numbers approx. 50 000 individuals; [30]) is marked. A citizen science network of greater than 2000 observers across Europe regularly report marked individuals, allowing the locations of individuals to be repeatedly tracked within and across years [31]. Here, we use this range-wide tracking of an expanding (in space) and advancing (in time) migratory shorebird, to explore whether individual plasticity or generational shifts are likely to have caused these changes in space and time, and to consider the evidence for potential developmental, environmental and demographic drivers of such changes.

## 2. Methods

### (a) Phenological change: repeated measures of individual spring arrival dates

**Figure 1.**
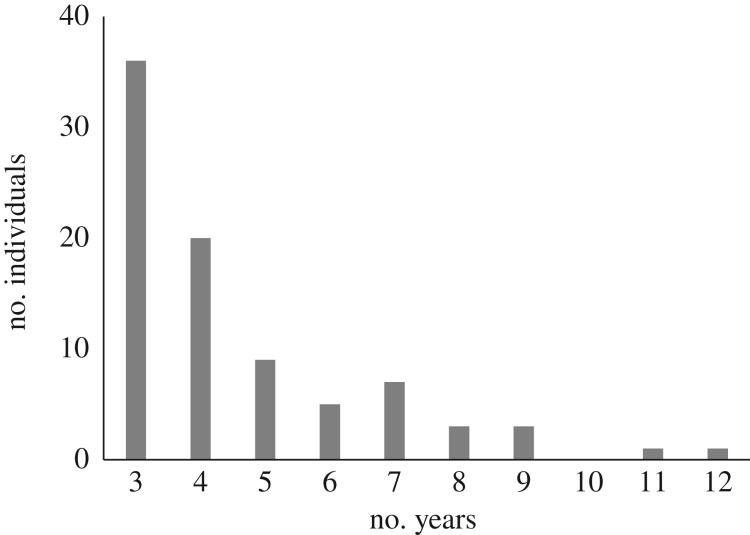
Number of years on which 85 individually marked Icelandic godwits have been recorded on spring arrival in Iceland, between 1999 and 2018.

Black-tailed godwits return to Iceland between mid-April and mid-May and, on arrival, flocks congregate on a small number of arrival sites [32]. Since 1999, standardized surveys of arrival sites in south and west Iceland have taken place from mid-April to early May, with the identities of all individually marked birds at all study locations being recorded every 1–3 days [13,32]. Between 1999 and 2018, arrival dates were recorded in at least 3 years for 85 individuals (figure 1). In order to quantify (a) the trends in arrival dates of individuals, we constructed a generalized linear model (GLM) with each individual arrival date (in Julian days) as the dependent variable, and year and individual as fixed effects (an extension of the model reported in Gill *et al*. [13] for a smaller sample). In order to then quantify (b) whether individual trends in arrival dates have changed in magnitude or (c) whether the frequency of individuals with differing arrival dates has changed over the 16-year survey period, we then constructed two further GLMs with the (b) trend in arrival date and (c) mean arrival date for each individual as the dependent variable, and the year of first spring arrival (i.e. the first year in which each individual was observed on arrival in Iceland) observation as a fixed effect.

### (b) Range change: non-breeding locations of marked individuals

Locations of individually marked godwits across the migratory range have been recorded by a network of citizen scientists since the mid-1990s. Here, we use all recorded non-breeding locations (i.e. excluding records within Iceland) of 631 individuals marked at the main post-breeding moult location in east England during the autumns of 1995–2014, and the winter (mid-October to mid-February) records of the 419 of these individuals observed during that period. To assess the role of individual movement in driving the northward range expansion, we use these sightings to quantify the total number of non-breeding locations (individual estuaries and wetlands), regions and countries (table 1) used by individuals tracked for differing numbers of years. To quantify the contribution of generational shifts in the frequency of individuals using different locations to the northwards range expansion, we then use these sightings to assess whether individuals marked in more recent years (which will be younger, on average, than previously marked individuals) are more likely to use recently colonized locations. We constructed GLMs with a binomial structure and a logit link, with the number of individuals marked in consecutive 5-year time periods since 1995 that winter in sites (i) colonized before or after the 1960s and (ii) north and south of 52° N (most of the recently colonized sites are north of 52° N; [31,33]) as the dependent variable, and time period as a fixed effect.

**Table 1.** The 121 winter locations across 26 regions and nine countries used by the individual godwits shown in figure 3. Regions in italics are colonized since the 1960s.

country	region	lat.–long.	no. of locations
N Ireland	*east*	54° N, 05° W	1
Ireland	*west*	53° N, 08° W	1
	*central*	52° N, 08° W	2
	*east*	53° N, 06° W	6
	south	51° N, 08° W	11
Wales	*north*	53° N, 03° W	2
	*west*	52° N, 04° W	1
England	*northwest*	53° N, 03° W	9
	*central*	52° N, 01° W	2
	*east*	52° N, 01° E	27
	south	50° N, 01° W	10
	*southeast*	51° N, 01° E	3
	*southwest*	50° N, 03° W	7
The Netherlands	*north*	53° N, 06° E	1
	*central*	52° N, 06° E	2
	*west*	52° N, 04° E	3
France	north	48° N, 01° W	3
	*northwest*	47° N, 02° W	7
	west	46° N, 01° W	10
Portugal	south	37° N, 08° W	4
	west	38° N, 09° W	3
Spain	*north*	43° N, 03° W	1
	*northwest*	42° N, 08° W	1
	*west*	38° N, 06° W	1
	south	37° N, 06° W	2
Morocco	west	30° N, 09° W	1

## 3. Results

### (a) Mechanisms driving phenological change: individual plasticity or generational shifts

**Figure 2.**
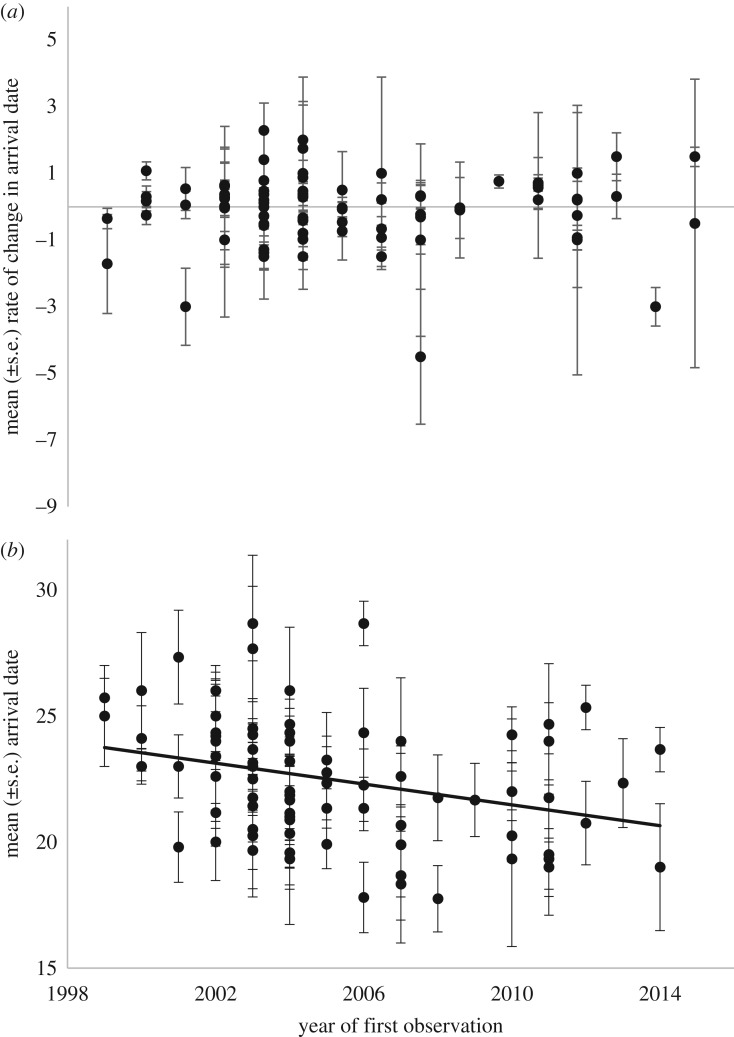
Changes in the mean (*a*) annual change in arrival date and (*b*) arrival date (days since 1 April) in Iceland of 85 individual godwits with the year in which they were first observed on arrival in Iceland (see table 2c for model details).

Although first spring observations of godwits in south Iceland have advanced by more than two weeks in the last two decades [24], trends in arrival dates of 85 marked individuals over the last 16 years vary significantly among individuals but show no consistent advances in individual arrival date (table 2a), and no change in individual arrival trends over the survey period (figure 2*a* and table 2b). However, the distribution of arrival dates of marked individuals has changed over time, with individuals first observed in more recent years tending to arrive earlier than individuals first recorded in the late 1990s/early 2000s (figure 2*b* and table 2c). Thus, while the arrival dates of individuals have not advanced, the frequency of early-arriving individuals has increased over this time period.

**Table 2.** Results of GLMs of (*a*) annual and individual variation in arrival dates of 85 godwits (3–12 years between 1999 and 2018) and variation in (*b*) annual trends in arrival dates and (*c*) mean arrival dates, in relation to the year in which those 85 individuals were first observed on arrival in Iceland.

	d.f.	*F*	*p*-value	slope (±s.e.)
(a)				
year	1	0.99	0.32	0.062 ± 0.06
individual	84	2.06	0.001	
error	300			
(b)				
first observation year	1	0.02	0.89	0.004 ± 0.03
error	83			
(c)				
first observation year	1	8.83	0.004	−0.21 ± 0.07
error	83			

### (b) Mechanisms driving range change: individual plasticity or generational shifts

**Figure 3.**
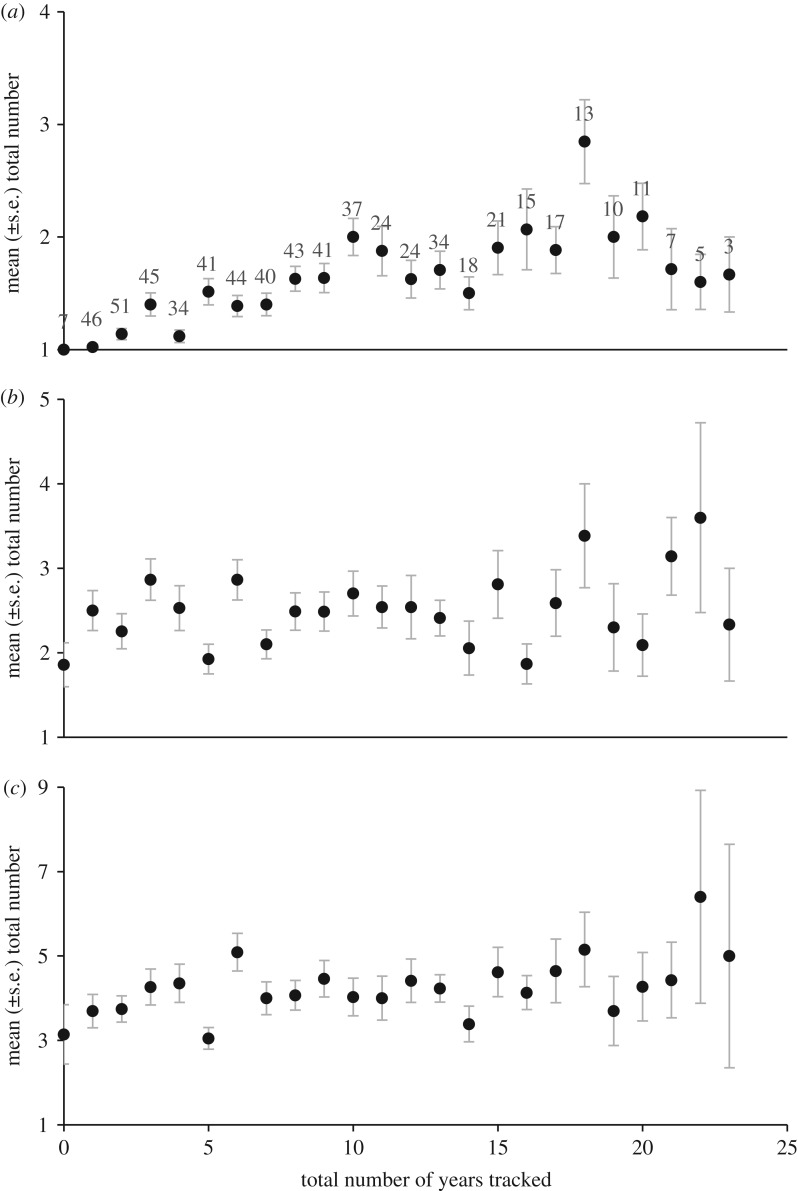
The total number of (*a*) countries, (*b*) regions and (*c*) locations on which individual godwits have ever been recorded in the total number of years over which each has been tracked. The number of individuals tracked for each total number of years is shown above the bars in (*a*); see table 1 for numbers of locations, regions and countries.

**Figure 4.**
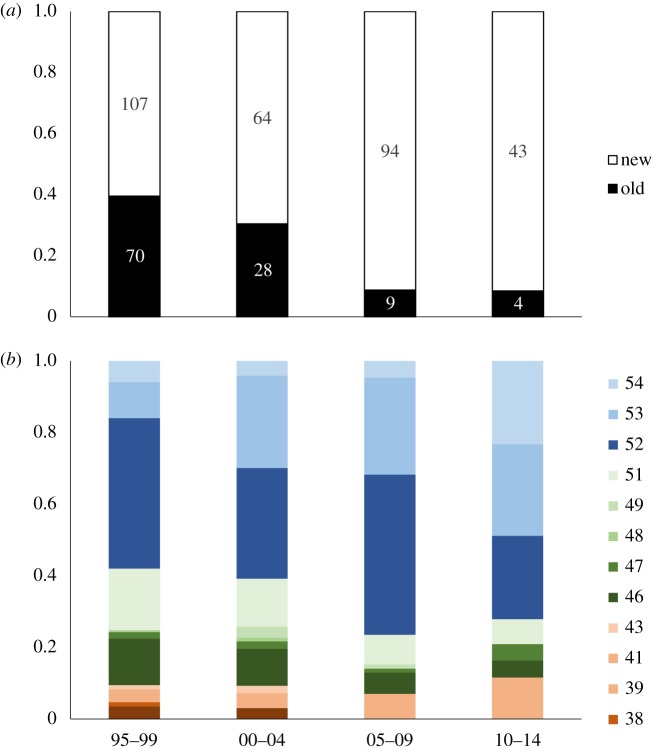
Changes through consecutive 5-year time intervals in the (*a*) proportion of individual godwits wintering in locations that have been occupied since the 1960s (old) or colonized since then (new), and (*b*) latitude of those winter locations. Numbers observed in each time period are shown in (*a*).

Repeated tracking of individual godwits across the migratory range for up to 23 years indicates a remarkably low level of individual plasticity in site use throughout their lives. On average, individuals are recorded on a total of only approximately 4 (±2.5 s.e.) non-breeding locations, regardless of the number of years for which they have been tracked (figure 3). In addition, these few locations are spread across, on average, 2.5 (±1.5) regions in 1.5 (±0.8) countries. Individuals are therefore highly restricted and consistent in their use of a small number of passage and winter locations, and these can be spread across the migratory range. However, over the two decades of continuous marking and tracking, with newly marked individuals being added to the population each year, the relative frequency of individuals using ‘new’ (colonized since the 1960s, table 3) winter sites has increased (figure 4*a*), and this is primarily through increased numbers of individuals using more northerly sites (figure 4*b* and table 3).

**Table 3.** Results of binomial models of variation through consecutive time intervals in the frequency of individually marked godwits recorded in winter locations (a) occupied since the 1960s (old, 0) or colonized since then (new, 1), and (b) north (0) and south (1) of 52° N.

	estimate	s.e.	*p*-value	odds ratio
(a)				
intercept	0.4	0.25		
year	−0.77	0.13	0.001	0.46
(b)				
intercept	−0.008	0.23		
year	−0.29	0.11	0.006	0.74

## 4. Discussion

The spring arrival dates of godwits in Iceland have advanced [24] and the breeding population has expanded northwards in recent decades, with rapid population increases in more northerly non-breeding locations [21]. Repeated tracking of individuals in space and time over this period has shown that these expansions and advances are driven by generational shifts in the frequency of individuals occupying different locations and migrating at different times, and not by individual plasticity. Individuals show lifelong consistency in the use of a small number of non-breeding locations, but the proportion of individuals occupying recently colonized sites is greater for more recently ringed birds. As recently colonized sites are primarily (but not exclusively) in the north of the range, and as the network of observers recording individual locations has been in operation throughout the 20-year survey period, the recorded shift in distribution is unlikely to be influenced by changes in reporting rates across the range.

The widely reported changes in phenology and distribution of many migratory species in recent decades may therefore be likely to result from generational shifts in the frequency of individuals with differing phenologies and using different locations. Generational shifts have been shown to drive shifts in distribution in other migratory systems. For example, a shift in the use of spring passage sites by a migratory wader population (continental black-tailed godwits, *Limosa limosa limosa*) was driven by new recruits to the population, while adults in the population continued to use the site they had always previously occupied [33]. Quantifying the role of generational shifts requires long-term tracking of individuals, and relatively few studies have this information during periods of change. However, tracking studies are increasingly being conducted, and studies tracking individuals over multiple years are typically reporting high levels of repeatability of individual timings and destinations [13–20], suggesting that the benefits of philopatry in the use of space and time are very strong [34,35]. Thus, generational shifts may be the primary mechanism through which phenological shifts and range change occur.

Identifying the environmental and demographic processes that drive generational shifts in space and time is therefore likely to be a critical step in understanding population-level responses to environmental change and the associated implications for conservation. Generational shifts in distribution or phenology could potentially arise through processes occurring at the following points in early life.

### (a) Developmental drivers of generational shifts

Conditions experienced at the natal stage, such as timing of hatching and/or conditions for growth and development, could influence the probability of those individuals undertaking different subsequent migratory routes and timings. For example, individuals hatched late in the season and/or with insufficient resources to fuel rapid growth are likely to migrate later, on average, and may thus have less time to locate more distant non-breeding locations. In such a case, an increase in the number of later-fledging individuals could drive recruitment into non-breeding locations that are closer to the breeding grounds. Similarly, natal conditions could potentially influence subsequent migration phenology, either directly through impacts on individual condition or indirectly through impacts on the conditions and potential flockmates encountered during the non-breeding season.

An important aspect of natal conditions that could potentially influence juvenile distribution and migratory timings is the changes in breeding phenology that are widely reported in many migratory populations [9,36]. Advances in nesting dates have been reported in many species and individual plasticity in nesting dates is common [37,38]. Thus, current evidence suggests that individual timings of arrival of migrants on breeding grounds tend to be consistent, but subsequent timings of breeding can vary greatly, and often vary in response to local weather conditions [6,39,40]. This changing phenology of natal conditions could potentially drive changes in subsequent phenology and distribution of new recruits to the population, if timing of fledging influences subsequent migratory routes and timings. A key mechanism that could drive such links is the potential for timing of fledging to influence the likely flockmates on migratory journeys, and the destinations to which they are travelling. For example, tracking of adult and juvenile lesser spotted eagles, *Clanga pomarina*, on their migratory journeys has shown that juveniles that departed at the same time as adults were significantly more likely to take the same routes as adults, and to have higher subsequent survival rates, than juveniles who departed without adults [41]. Timing-driven access to social cues in migratory flocks could therefore be an important driver of the migratory routes and destinations located by juveniles, and changes in the timing of fledging could therefore drive changes in the non-breeding distribution of migratory species.

### (b) Environmental effects on generational shifts

Changing environmental conditions could directly influence the probability of recruits migrating at specific times or locating specific non-breeding locations. For example, changing weather conditions (e.g. windspeeds or directions) could alter the proportions of recruits migrating at different times or taking different routes. However, while wind conditions can have important effects on migrating birds [42], individual consistency in migratory destinations and timings would mean that such effects could only operate in early life (i.e. during settlement/recruitment).

### (c) Demographic effects on generational shifts

Disproportionate changes in survival rates of recruits that differ in distribution or timing could lead to generational shifts. For example, while the numbers of individuals recruiting into more northerly winter locations or arriving early on the breeding grounds may not be changing, those individuals could be increasingly likely to survive, for example, as a consequence of ameliorating weather conditions in northerly areas or on arrival in the breeding areas. Changing patterns of survival may be particularly relevant in systems with range expansion into areas in which weather conditions are changing as a consequence of climatic change [43], and these effects could operate alongside developmental or environmental effects.

### (d) Drivers of generational shifts in Icelandic godwits

In Icelandic black-tailed godwits, rapid warming has occurred on the breeding grounds in recent decades, and nesting dates are earlier [6] and productivity is higher [26] in warmer years. This warming-driven increase in productivity is likely to have fuelled the colonization of colder breeding areas in the north, where nest-laying and hatching dates are, on average, later than in more southerly breeding areas [6]. Individuals from these colder and more recently colonized breeding areas are more likely to also winter in the more recently colonized non-breeding areas [31], and these seasonal links could thus result from regional-scale differences in the timing of fledging and subsequent social cues available to juveniles undertaking their first migration.

Warming-driven advances in nesting dates could also have driven the advancing spring arrival of godwits in Iceland, as previous analyses have shown that (i) individuals wintering in more southerly areas of Europe [29] and breeding in the warmer areas of south and west Iceland [32] arrive first, and (ii) more recently hatched individuals tend to have earlier spring arrival dates than individuals hatched in the 1990s [13]. This suggests that the generational shifts driving the phenological advance in this system (figure 2) could potentially result from increased numbers of early-hatched individuals from the traditionally occupied areas of Iceland that, because of their early fledging, are also more likely to have the time, condition and social cues to both locate traditionally occupied winter areas and return early in spring. Increased survival rates of early-arrivers and northerly winterers could also be contributing to these changes in space and time.

### (e) Climate change and generational shifts in space and time

If climate-driven shifts in breeding phenology can alter the frequency of juveniles undertaking different migratory routes, destinations and timings, this could be an important route through which climate-associated shifts in range and phenology are manifest. A common pattern among migratory species at present is those migrating longer distances are less likely to show shifts in migration phenology [44,45]. As longer-distance migrants typically arrive later on the breeding grounds and have a smaller gap between arrival and laying than shorter-distance migrants [13], they may have a more limited capacity to alter breeding phenology (and thus generational shifts resulting from shifts in breeding phenology are less likely to occur). The effects of climate warming on breeding phenology can thus have potentially far-reaching consequences for migratory populations.

### (f) Future research

Identifying the contribution of developmental, environmental and demographic change to generational shifts, and the conditions in which each might be most relevant, will require studies in which the effects of natal conditions, environmental conditions experienced by juvenile individuals undertaking different migratory routes and timings, and the demographic consequences of conditions experienced at destinations can be measured. Tracking individuals from fledging to recruitment into adulthood is difficult because survival rates at this life stage are typically low, and retrieval of tags can be challenging because the subsequent breeding locations of these individuals is typically unknown, but technological advances will hopefully make these issues more tractable in the near future. Long-term studies of seasonal patterns of nest loss, timing of replacement clutches and juvenile fledging will also be particularly valuable in identifying the potential role of breeding phenology in driving change in migratory systems. Quantifying the developmental, environmental and demographic processes that influence individual migratory routes, destinations and timings will be key to understanding future rates and directions of spatial and phenological change in migratory species, and the associated implications for designing effective protected area networks and conservation actions for these species.

## Data accessibility

All data are presented and accessible in the figures.

## References

[1] Vickery JA, Ewing SR, Smith KW, Pain DJ, Bairlein F, Škorpilová J, Gregory RD 2014 The decline of Afro-Palaearctic migrants and an assessment of potential causes. Ibis 156, 1–22. (10.1111/ibi.12118)

[2] Gilroy JJ, Gill JA, Butchart SH, Jones VR, Franco AM 2016 Migratory diversity predicts population declines in birds. Ecol. Lett. 19, 308–317. (10.1111/ele.12569) 26807694

[3] Studds CE et al. 2017 Rapid population decline in migratory shorebirds relying on Yellow Sea tidal mudflats as stopover sites. Nat. Commun. 8, 14895 (10.1038/ncomms14895) 28406155 PMC5399291

[4] Runge CA, Watson JE, Butchart SH, Hanson JO, Possingham HP, Fuller RA 2015 Protected areas and global conservation of migratory birds. Science 350, 1255–1258. (10.1126/science.aac9180) 26785490

[5] Méndez V, Gill JA, Alves JA, Burton NH, Davies RG 2018 Consequences of population change for local abundance and site occupancy of wintering waterbirds. Divers. Distrib. 24, 24–35. (10.1111/ddi.12653)

[6] Alves JA, Gunnarsson TG, Sutherland WJ, Potts PM, Gill JA 2019 Linking warming effects on phenology and demography with range expansion in a migratory bird population. Ecol. Evol. 9, 2365–2375. (10.1002/ece3.4746) 30891186 PMC6405501

[7] Møller AP, Rubolini D, Lehikoinen E 2008 Populations of migratory bird species that did not show a phenological response to climate change are declining. Proc. Natl Acad. Sci. USA 105, 16 195–16 200. (10.1073/pnas.0803825105) PMC2571031 18849475

[8] Miller-Rushing AJ, Høye TT, Inouye DW, Post E 2010 The effects of phenological mismatches on demography. Phil. Trans. R. Soc. B 365, 3177–3186. (10.1098/rstb.2010.0148) 20819811 PMC2981949

[9] Knudsen E et al. 2011 Challenging claims in the study of migratory birds and climate change. Biol. Rev. 86, 928–946 (10.1111/j.1469-185X.2011.00179.x) 21489123

[10] La Sorte FA, Thompson FR. 2007 Poleward shifts in winter ranges of North American birds. Ecology 88, 1803–1812. (10.1890/06-1072.1) 17645026

[11] Virkkala R, Lehikoinen A 2014 Patterns of climate-induced density shifts of species: poleward shifts faster in northern boreal birds than in southern birds. Glob. Change Biol. 20, 2995–3003. (10.1111/gcb.12573) 24729475

[12] Sanderson FJ et al. 2016 Assessing the performance of EU nature legislation in protecting target bird species in an era of climate change. Conserv. Lett. 9, 172–180. (10.1111/conl.12196)

[13] Gill JA, Alves JA, Sutherland WJ, Appleton GF, Potts PM, Gunnarsson TG 2014 Why is timing of bird migration advancing when individuals are not? Proc. R. Soc. B 281, 20132161 (10.1098/rspb.2013.2161) PMC3843828 24225454

[14] Vardanis Y, Klaassen RH, Strandberg R, Alerstam T 2011 Individuality in bird migration: routes and timing. Biol. Lett. 7, 20101180 (10.1098/rsbl.2010.1180) PMC3130220 21307045

[15] Stanley CQ, MacPherson M, Fraser KC, McKinnon EA, Stutchbury BJ 2012 Repeat tracking of individual songbirds reveals consistent migration timing but flexibility in route. PLoS ONE 7, e40688 (10.1371/journal.pone.0040688) 22848395 PMC3405083

[16] Conklin JR, Battley PF, Potter MA 2013 Absolute consistency: individual versus population variation in annual-cycle schedules of a long-distance migrant bird. PLoS ONE 8, e54535 (10.1371/journal.pone.0054535) 23342168 PMC3546993

[17] López-López P, García-Ripollés C, Urios V 2014 Individual repeatability in timing and spatial flexibility of migration routes of trans-Saharan migratory raptors. Curr. Zool. 60, 642–652. (10.1093/czoolo/60.5.642) PMC5804231 29491895

[18] Lourenço PM, Alves JA, Reneerkens J, Loonstra J, Potts PM, Granadeiro JP, Catry T 2016 Influence of age and sex on winter site fidelity of sanderlings *Calidris alba*. PeerJ 4, e2517 (10.7717/peerj.2517) 27703860 PMC5045889

[19] Hasselquist D, Montràs-Janer T, Tarka M, Hansson B 2017 Individual consistency of long-distance migration in a songbird: significant repeatability of autumn route, stopovers and wintering sites but not in timing of migration. J. Avian Biol. 48, 91–102. (10.1111/jav.01292)

[20] Pedersen L, Jackson K, Thorup K, Tøttrup AP 2018 Full-year tracking suggests endogenous control of migration timing in a long-distance migratory songbird. Behav. Ecol. Sociobiol. 72, 139 (10.1007/s00265-018-2553-z)

[21] Gill JA, Norris K, Potts PM, Gunnarsson TG, Atkinson PW, Sutherland WJ 2001 The buffer effect and large-scale population regulation in migratory birds. Nature 412, 436 (10.1038/35086568) 11473317

[22] Gunnarsson TG, Gill JA, Sigurbjörnsson T, Sutherland WJ 2004 Pair bonds: arrival synchrony in migratory birds. Nature 431, 646 (10.1038/431646a) 15470417

[23] Alves JA, Gunnarsson TG, Hayhow DB, Appleton GF, Potts PM, Sutherland WJ, Gill JA 2013 Costs, benefits, and fitness consequences of different migratory strategies. Ecology 94, 11–17. (10.1890/12-0737.1) 23600235

[24] Gunnarsson TG, Tómasson G 2011 Flexibility in spring arrival of migratory birds at northern latitudes under rapid temperature changes. Bird Study 58, 1–12. (10.1080/00063657.2010.526999)

[25] Gunnarsson TG, Gill JA, Appleton GF, Gíslason H, Gardarsson A, Watkinson AR, Sutherland WJ 2006 Large-scale habitat associations of birds in lowland Iceland: implications for conservation. Biol. Conserv. 128, 265–275. (10.1016/j.biocon.2005.09.034)

[26] Gunnarsson TG, Jóhannesdóttir L, Alves JA, Þórisson B, Gill JA 2017 Effects of spring temperature and volcanic eruptions on wader productivity. Ibis 159, 467–471. (10.1111/ibi.12449)

[27] Gunnarsson TG, Gill JA, Petersen A, Appleton GF, Sutherland WJ 2005 A double buffer effect in a migratory shorebird population. J. Anim. Ecol. 74, 965–971. (10.1111/j.1365-2656.2005.00994.x)

[28] Prater AJ 1975 The wintering population of the Black-tailed Godwit. Bird Study 22, 169–176. (10.1080/00063657509476461)

[29] Alves JA, Gunnarsson TG, Potts PM, Gélinaud G, Sutherland WJ, Gill JA 2012 Overtaking on migration: does longer distance migration always incur a penalty? Oikos 121, 464–470. (10.1111/j.1600-0706.2011.19678.x)

[30] Gunnarsson TG, Gill JA, Potts PM, Atkinson PW, Croger RE, Gélinaud G, Gardarsson A, Sutherland WJ 2005 Estimating population size in black-tailed godwits *Limosa limosa islandica* by colour-marking. Bird Study 52, 153–158. (10.1080/00063650509461385)

[31] Gunnarsson TG, Gill JA, Newton J, Potts PM, Sutherland WJ 2005 Seasonal matching of habitat quality and fitness in a migratory bird. Proc. R. Soc. Lond. B 272, 2319–2323. (10.1098/rspb.2005.3214) PMC1560186 16191646

[32] Gunnarsson TG, Gill JA, Atkinson PW, Gelinaud G, Potts PM, Croger RE, Gudmundsson GA, Appleton GF, Sutherland WJ 2006 Population-scale drivers of individual arrival times in migratory birds. J. Anim. Ecol. 75, 1119–1127. (10.1111/j.1365-2656.2006.01131.x) 16922847

[33] Verhoeven MA, Loonstra AJ, Hooijmeijer JC, Masero JA, Piersma T, Senner NR 2018 Generational shift in spring staging site use by a long-distance migratory bird. Biol. Lett. 14, 20170663 (10.1098/rsbl.2017.0663) 29445041 PMC5830661

[34] Kokko H, Sutherland WJ 2001 Ecological traps in changing environments: ecological and evolutionary consequences of a behaviourally mediated Allee effect. Evol. Ecol. Res. 3, 603–610.

[35] Winger BM, Auteri GG, Pegan TM, Weeks BC 2018 A long winter for the Red Queen: rethinking the evolution of seasonal migration. Biol. Rev. 94, 737–752. (10.1111/brv.12476) 30393938

[36] Dunn PO, Møller AP 2014 Changes in breeding phenology and population size of birds. J. Anim. Ecol. 83, 729–739. (10.1111/1365-2656.12162) 24117440

[37] Visser ME, te Marvelde L, Lof ME 2012 Adaptive phenological mismatches of birds and their food in a warming world. J. Ornithol. 153, 75–84. (10.1007/s10336-011-0770-6)

[38] Charmantier A, Gienapp P 2014 Climate change and timing of avian breeding and migration: evolutionary versus plastic changes. Evol. Appl. 7, 15–28. (10.1111/eva.12126) 24454545 PMC3894895

[39] Townsend AK, Sillett TS, Lany NK, Kaiser SA, Rodenhouse NL, Webster MS, Holmes RT 2013 Warm springs, early lay dates, and double brooding in a North American migratory songbird, the black-throated blue warbler. PLoS ONE 8, e59467 (10.1371/journal.pone.0059467) 23565154 PMC3614938

[40] Visser ME, Gienapp P, Husby A, Morrisey M, de la Hera I, Pulido F, Both C. 2015 Effects of spring temperatures on the strength of selection on timing of reproduction in a long-distance migratory bird. PLoS Biol. 13, e1002120 (10.1371/journal.pbio.1002120) 25848856 PMC4388467

[41] Meyburg BU, Bergmanis U, Langgemach T, Graszynski K, Hinz A, Börner I, Meyburg C, Vansteelant WM 2017 Orientation of native versus translocated juvenile lesser spotted eagles (*Clanga pomarina*) on the first autumn migration. J. Exp. Biol. 220, 2765–2776. (10.1242/jeb.148932) 28768749 PMC5558239

[42] Shamoun-Baranes J, Leyrer J, van Loon E, Bocher P, Robin F, Meunier F, Piersma T. 2010 Stochastic atmospheric assistance and the use of emergency staging sites by migrants. Proc. R. Soc. B 277, 20092112 (10.1098/rspb.2009.2112) PMC2871836 20071381

[43] McKechnie AE, Wolf BO 2010 Climate change increases the likelihood of catastrophic avian mortality events during extreme heat waves. Biol. Lett. 6, 253–256. (10.1098/rsbl.2009.0702) 19793742 PMC2865035

[44] Butler CJ 2003 The disproportionate effect of global warming on the arrival dates of short-distance migratory birds in North America. Ibis 145, 484–495. (10.1046/j.1474-919X.2003.00193.x)

[45] Rubolini D, Møller AP, Rainio K, Lehikoinen E 2007 Intraspecific consistency and geographic variability in temporal trends of spring migration phenology among European bird species. Clim. Res. 35, 135–146. (10.3354/cr00720)

